# Biotechnological Processes in Fruit Vinegar Production

**DOI:** 10.3390/foods10050945

**Published:** 2021-04-26

**Authors:** Luz María Luzón-Quintana, Remedios Castro, Enrique Durán-Guerrero

**Affiliations:** Analytical Chemistry Department, Faculty of Sciences-IVAGRO, Agrifood Campus of International Excellence (ceiA3), University of Cadiz, Campus Universitario de Puerto Real, s/n, Puerto Real, 11510 Cadiz, Spain; luzmaria.luzonquintana@alum.uca.es (L.M.L.-Q.); enrique.duranguerrero@uca.es (E.D.-G.)

**Keywords:** vinegar, fruit, fermentation, acetic acid bacteria, submerged culture, surface culture

## Abstract

The production of fruit vinegars as a way of making use of fruit by-products is an option widely used by the food industry, since surplus or second quality fruit can be used without compromising the quality of the final product. The acetic nature of vinegars and its subsequent impact on the organoleptic properties of the final product allows almost any type of fruit to be used for its elaboration. A growing number of scientific research studies are being carried out on this matrix, and they are revealing the importance of controlling the processes involved in vinegar elaboration. Thus, in this review, we will deal with the incidence of technological and biotechnological processes on the elaboration of fruit vinegars other than grapes. The preparation and production of the juice for the elaboration of the vinegar by means of different procedures is an essential step for the final quality of the product, among which crushing or pressing are the most employed. The different conditions and processing methods of both alcoholic and acetic fermentation also affect significantly the final characteristics of the vinegar produced. For the alcoholic fermentation, the choice between spontaneous or inoculated procedure, together with the microorganisms present in the process, have special relevance. For the acetic fermentation, the type of acetification system employed (surface or submerged) is one of the most influential factors for the final physicochemical properties of fruit vinegars. Some promising research lines regarding fruit vinegar production are the use of commercial initiators to start the acetic fermentation, the use of thermotolerant bacteria that would allow acetic fermentation to be carried out at higher temperatures, or the use of innovative technologies such as high hydrostatic pressure, ultrasound, microwaves, pulsed electric fields, and so on, to obtain high-quality fruit vinegars.

## 1. Introduction

Vinegar has been part of the human diet since ancient times and has been widely used as a preservative, condiment, aromatizer, and even as a healthy drink. Moreover, it has also been traditionally used in ancient medicine because of its medicinal properties [1]. Vinegar can be made from any carbohydrate source, amylaceous, or sugary substrate through two successive fermentations: alcoholic fermentation, which is carried out by means of yeasts, and acetic fermentation, with acetic bacteria as the protagonist.

Every year, large amounts of fruit are produced and wasted since the excess cannot be consumed or because the fruits are considered of a second or third quality category. According to the FAO [2], 21.6% of the fruit produced in the world is wasted, starting from the post-harvest stage until its distribution. Very often, fruit is rejected simply because of its “imperfect” appearance or inadequate size, even if the fruit is perfectly edible. It is true that although there are alternatives such as the production of fruit purees, juices, or even fruit jams, large quantities are still wasted as the fruit is left in the fields until it decomposes or is immediately disposed of as waste. These actions lead to both ecological and economic problems; therefore, environmental pollution and rising prices can be the consequence of fruit overproduction. Hence, alternatives that can use this surplus and thus reduce the impact generated on the fruit industry are extremely valuable. Some possible options, related to the vinegar industry, could be the maceration of fruits with vinegar, the enrichment of vinegars with fruit fiber, or the employment of fruits for vinegar production.

As it has been mentioned, one of the possible uses of fruit industry residues is the elaboration of macerated vinegars using different parts of fruit. The peels of citrus fruits such as orange, lemon, lime, grapefruit, or the entire strawberry have been employed several times for the maceration with vinegar [3,4,5]. Some examples of vinegar maceration with other fruits such as banana, passion fruit, or apple have also been found in the literature [6].

Maceration is not the only way to make use of fruit waste. The dietary fiber extracted from these wastes can be used to enrich other foods. The dietetic fiber derived from fruits is increasingly introduced in the market these days because of its higher nutritional quality compared to the dietetic fiber derived from cereals. Several authors have studied dietary fiber from orange, lemon, lime, grapefruit, or apple peels [7,8,9,10]. Other authors [11] studied the enrichment of vinegar with dietary fibers from orange and lemon, and it was observed that with that enrichment, the volatile and polyphenolic compounds contained in the starting vinegar were enhanced, among which the orange fiber was the one that provided the highest content in volatiles and the lemon fiber was the one that provided the highest content in polyphenols.

Another option to exploit this surplus fruit would be the production of vinegars directly from them. Although the most commonly consumed vinegar in the world is wine vinegar (from grapes), there are many types of vinegars according to the raw material used for their production. Some of the most important examples are rice or sake vinegar, malt vinegar, cider vinegar, or general fruit vinegars other than grapes. Nowadays, a really popular vinegar is apple vinegar, which seems to have considerable healthy benefits, such as weight loss, the lowering of the blood glucose levels in people with type 2 diabetes mellitus, or the lowering of the risk of heart diseases, among others [12].

The production of fruit vinegars as a way of making use of fruit by-products is widely employed by the food industry, since it allows them to exploit surplus and second-quality fruit without compromising the quality of the final product. The acetic nature of fruit vinegars and the high sensory impact that this acid produces on the organoleptic properties of the product allow almost any type of fruit to be used for its elaboration.

**Figure 1:**
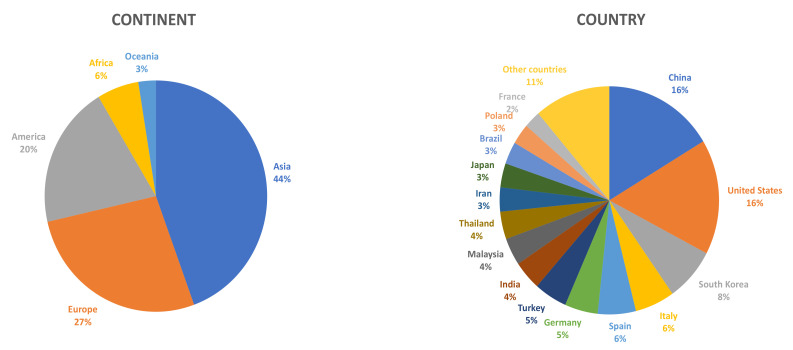
Percent distribution of scientific articles on fruit vinegar published from 2015 to 2020, according to the origin of the research groups (continent/country) (Source: Scopus).

Although Asian countries were the first ones to become interested in this type of product, more and more scientific research is being carried out in other parts of the world on this matrix (Figure 1).

**Figure 2:**
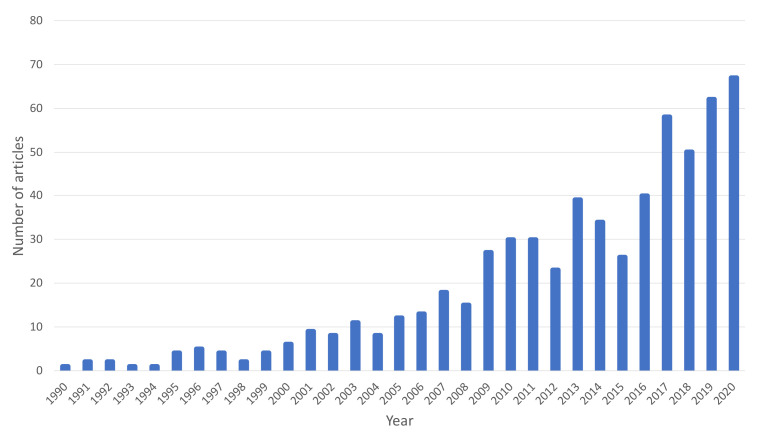
Number of scientific articles on fruit vinegar published per year (Source: Scopus).

Figure 2 shows the distribution of the number of scientific articles on fruit vinegars published between 1990 and 2020. As can be seen, there has been an exponential growth in recent years, which would demonstrate the growing interest of the scientific community in this type of product.

**Figure 3:**
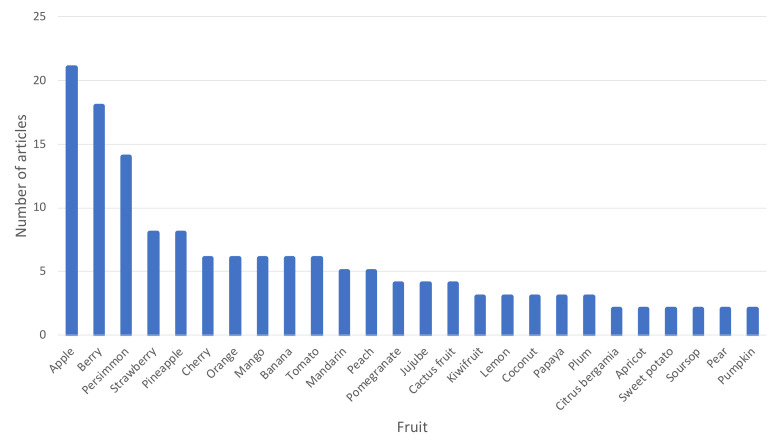
Different fruits (other than grapes) employed for the elaboration of fruit vinegars for which two or more scientific articles about the technological process have been found in the literature from 1990 to 2020.

With regard to the production process of fruit vinegars in particular, Figure 3 shows the different fruits for which two or more research studies have been found from 1990 to date for the production of fruit vinegars. It can be seen that many fruits have been explored for the elaboration of vinegars, the most common being apple, different berries, persimmon, strawberry, pineapple, cherry, orange, mango, or banana, among others. This figure does not include grapes as fruit, since this would require the inclusion of all the references to wine vinegars, which is not the object of this scientific review.

This increasing tendency has enabled that some problems related to the authentication of fruit vinegars on the raw material, the elaboration process, or the geographical origin, have started [13]. The quality of fruit vinegar is related to the amount of some specific bioactive compounds. The addition of cheaper substitutes or the total substitution of these particular compounds, which define the quality of highly recognized vinegars, together with the possible use of false labeling, are usual authentication problems in the case of fruit vinegars. The different authentication methodologies used for the specific case of this type of product can be found in the bibliography. Unambiguous constituents [14,15], several molecular isotope ratios [16,17], spectroscopic techniques, such as infrared and fluorescence spectroscopy in combination with several chemometric techniques [13], and even electronic nose and electronic tongue [18] have been used to detect adulteration, mainly related to raw material and/or geographical origin.

Therefore, this bibliographic review will address the influence of technological and biotechnological processes on the production of fruit vinegars other than grapes. On the one hand, it will analyze the different juice preparation and extraction procedures such as the crushing, grinding, or peeling of the fruit. The different conditions and methods applied to both the alcoholic and the acetic fermentation will also be studied, as well as the different microorganisms responsible for each process along with the physicochemical properties of the final products.

## 2. Raw Material

The raw material employed for vinegar production plays an important role in the final characteristics of the developed product. As it was mentioned in the Introduction section, although grape is the most employed fruit for vinegar production, due to the long tradition of winemaking, many other fruits have been employed for the production of vinegar (Figure 3). Fruits are well known to contain multiple bioactive and healthy compounds, such as amino acids, organic acids, phenolics, vitamins, and mineral substances [19]. However, not all fruits present the same amount of these kinds of compounds, and there also could be significant differences in the same type of fruit. For example, different values of organic acids have been found in orange juices (malic acid: 0.000–4.294 g/L; citric acid: 1.471–12.525 g/L; succinic acid: 0.000–0.229 g/L; tartaric acid: 0.000–0.363 g/L), pineapple juices (malic acid: 0.291–3.505 g/L; citric acid: 0.000–9.911 g/L), or apple juices (lactic acid: 0.000–0.267 g/L; malic acid: 0.225–4.907 g/L; citric acid: 0.145–3.699 g/L) [20]. Regarding the phenolic content and antioxidant activity, Sun et al. reported some values in common fruits [21]. Cranberry had the highest total phenolic content (527.2 mg/100 g, expressed as mg of gallic acid equivalent/100 g of fresh weight of the edible part of fruits), followed by apple (296.3 mg/100 g), red grape (201.0 mg/100 g), strawberry (160.0 mg/100 g), pineapple (94.3 mg/100 g), banana (90.4 mg/100 g), peach (84.6 mg/100 g), lemon (81.9 mg/100 g), orange (81.2 mg/100 g), pear (70.6 mg/100 g), and grapefruit (49.6 mg/100 g). Regarding the antioxidant activity, expressed as µmol of vitamin C equivalent/g of fresh weight of the edible part of fruits, cranberry had the highest activity (177.0 µmol/g), followed by apple (97.6 µmol/g), red grape (64.7 µmol/g), strawberry (64.4 µmol/g), peach (49.5 µmol/g), lemon (42.8 µmol/g), pear (34.2 µmol/g), banana (32.8 µmol/g), orange (31.5 µmol/g), grapefruit (24.7 µmol/g), and pineapple (16.9 µmol/g) [21]. Concerning the amino acid profile of fruits, they contain mostly the endogenous amino acids produced by the plant and some others produced by the microorganisms living on it. However, the products of technological treatments, such as wines and vinegars, show a much more complicated amino acid profile because of the fermentation processes. In particular, fermentation can both modify the concentration of existing amino acids and introduce new ones in the product [22]. The sugar content of fruits is also an important parameter to be taken into account when dealing with vinegar production, because it is directly related to the alcohol concentration that will be achieved during alcoholic fermentation, and therefore to the final acidity of the vinegar produced during acetic fermentation. Fructose, glucose, and sucrose are usually the most abundant sugars, but in some fruits such as pears, other sugars can be present in higher concentrations: e.g., sorbitol (27.39 g/100 g of dry matter (DM)) against glucose (11.74 g/100 g DM) [23]. The content of sugars can also vary depending on the fruit. For instance, the content of fructose determined by Chapman and Horvat was around 47.50 g/100 g DM in pear, but it was only 14.92 g/100 g DM in peach, and the sucrose level presented higher values in peach (53.60 g/100 g DM) than in pear (2.45 g/100 g DM) [23]. In addition, the volatile composition of fruits is important in the production of fruit vinegar because many factors affect this composition, including the genetic makeup, degree of maturity, environmental conditions, post-harvest handling, and storage. The main chemical families of volatile compounds in fruits are esters, alcohols, aldehydes, ketones, lactones, terpenoids, and apocarotenoids [24]. On the other hand, the original state of the fruit (fresh, canned, frozen) could also affect the content of bioactive compounds such as vitamins or polyphenols [25]. Therefore, it is expectable that the chemical composition of the initial fruit will affect the final properties of the vinegar. For example, the phenolic content of the vinegar has been proven to be directly related to the initial content of the raw material: whereas gallic acid was higher in grape vinegar, catechin was present in higher concentration in apple vinegar, and the same distribution was observed in the respective raw materials [26]. Similar conclusions were obtained by Sengun et al. who described that phenolic and flavonoid contents, together with other characteristics of fruit vinegars, such as total acidity, pH, brix, color, or antioxidant and antimicrobial capacities were also related to the raw material employed [19]. For example, the highest total phenolic content and total flavonoid content were found in the blackberry (1162 mg gallic acid equivalent (GAE)/L) and plum vinegar (470.86 mg catechin/L), respectively. Mandarin vinegar had the lowest levels in terms of total phenolic (933 mg GAE/L) and flavonoid content (66.64 mg catechin/L). Apricot and plum were the fruits that produced vinegars with higher antioxidant activities: apricot, DPPH (2,2-diphenyl-1-picrylhydrazyl) 0.1302 µg TE (Trolox equivalent)/mL, ABTS (2,2’-azino-bis(3-ethylbenzothiazoline-6-sulfonic acid)) 0.885 µg TE/mL; plum, DPPH 0.302 µg TE/mL, ABTS 0.538 µg TE/mL. It is interesting to note that grape vinegar presented one of the lowest values compared to the other fruits studied (DPPH 0.119 µg TE/mL, ABTS 0.441 µg TE/mL) [19]. This fact corroborates the importance of the employment of fruits other than grapes to produce vinegars with healthy benefits. Moreover, Coelho et al. [27] presented values of antioxidant activity, which are expressed as equivalents of Fe_2_SO_4_, of four fruit vinegars: 11.0 ± 1.67 mmol L^−1^ for orange, 4.8 ± 0.5 mmol L^−1^ for mango, 18.6 ± 2.33 mmol L^−1^ for cherry, and 3.7 ± 0.3 mmol L^−1^ for banana vinegar. These antioxidant activity values were close to the reported for the corresponding fruit, and they were between 8 and 40 folds higher than the one found in a commercial cider vinegar. This fact showed the possibility of preservation of functional features during vinegar making. However, Bakir et al. described significant decreases in the antioxidant activity observed from apple juice to apple vinegars [26]. Regarding the composition of volatile compounds in the vinegars obtained from different fruits, Coelho et al. described an insight of *Acetobacter* metabolism on aroma composition, but despite the transformations observed, fruit vinegars presented high contents of minor volatiles coherent with varietal aroma: monoterpenic alcohols for orange vinegar, C_13_-norisoprenoids and benzaldehyde for cherry vinegar, esters for banana vinegar, and furaneol for mango vinegar [27]. All this information corroborates the importance of the raw material on the characteristics of the final product.

## 3. Juice Extraction

There are several critical steps in the production of fruit vinegar, and each one of them requires special attention. The first important phase is the preparation and extraction of the juice from the raw material, since the composition of the same can be decisive regarding the organoleptic and bioactive properties as well as the quality of the vinegar as a final product.

The preparation of the raw material includes all the operations and processes that are necessary for the elaboration of protein and sugar solutions capable of being transformed by means of fermentation. Low-quality fruit, waste, or by-products generated during the cultivation of the same, as well as seasonal surpluses, are used for vinegar production [28,29,30,31,32,33,34,35]. In fact, as it has been indicated previously, fruits are rich sources of potentially bioactive substances such as flavonoids, stilbenes, vitamins, fiber, phenolic acids, coumarins, tannins, phytonutrients, etc. [36].

Although the acetification processes may vary slightly depending on the particular raw material and the type of vinegar produced, they are all essentially very similar. Most investigations coincide in that the raw material must first be washed with tap or chlorinated water, in order to remove surface dirt, impurities and any other possible traces of undesirable microorganisms or even pesticides [37,38,39,40,41,42,43,44,45]. Then, it must be thoroughly dried at room temperature for later use [37,39,46]. The treatment that the fruit receives afterwards will depend on each particular case.

Crushing is a very simple complementary single operation performed in most research studies [45,46,47,48,49,50]. It can be done either by mechanical juicer [42,50,51], blender [29,52,53], or food mixer [37,54,55,56,57]. In some occasions, the mixture may have water added as a final step [58,59] or it may be added as a preparation for the crushing [60]. Some authors, so as to prevent undesirable microbial growth, add sulfur dioxide to the juice [29,43,53,54,56,60,61], pasteurize the sample [51,62], or heat sterilize it [63]. To ensure an adequate final acidity in the resulting vinegar and to be able to determine the contribution of the sugar content to the properties of the vinegar, once the juice has been obtained, sucrose [34,37,56], glucose [64], white granulated sugar [38], bee honey [65], or heat concentrate can be added [55] with frequent agitation [66] or using an evaporator [40,44].

Cejudo-Bastante et al. [28], for the production of orange vinegar, added pectolytic enzymes as a clarifying agent and diammonium sulfate as a nutrient for the subsequent alcoholic fermentation of the orange juice. Other authors also employed pectolytic enzymes after the crushing of persimmon fruit [54] or strawberry [49] in order to facilitate the release of volatiles in addition to the clarification of the product. Hidalgo et al. [29], in the case of strawberry vinegar, used crushing as the main operation to obtain strawberry juice; however, they performed two different treatments: in one of them, they crushed the fruit pulp, and in the other one, they used juice obtained from a vertical press. Strawberry vinegar was produced only when the pulp was used. However, as it was somewhat dense, it had to be later pressed to remove solid waste and obtain a translucent juice. On the contrary, Dias et al. [43] assured that to improve the sedimentation of the non-fermentable solids, bentonite had to be added to the must. On the other hand, Su and Silva [67], to prepare blueberry vinegar, crushed and pressed the blueberries to obtain the starting juice. Then, the resulting pomace (those components from the raw material that had not been transformed into juice) was analyzed and presented a high content of polyphenols, both extractable and non-extractable. It is important to take into account that the skin of some berries is rich in phenolic compounds and that a conventional processing does not reflect all these components in the final product. Therefore, the improvement in the processing of the raw material is essential for the improvement of quality vinegar production [65].

Milling is also used to obtain fruit juice, which, together with filtration and other additional operations, are the physical stages of the individual operations. Tsen et al. [68] for the production of tomato vinegar ground the tomatoes in a mixer and then subjected them to a thermal treatment from 95 °C to below zero. Then, they were filtered to remove the skin bits and seeds. Once the tomato juice was obtained, it was thermally treated again to induce the deactivation of pectin methyl esterases, polygalacturonases, and peroxidases, which are responsible for the viscosity level of the fluid obtained [69]. On the other hand, Koh et al. [39] crushed the tomatoes and then filtered them using a depulper so that the tomato juice and the layer formed by the skin and seeds could be separated. These researchers also performed a thermal treatment of the juice at 100 °C for several time intervals in order to study the influence from temperature and time on °Brix, pH, total titratable acidity (TTA), and color properties. Then, it was observed how as the heating time was increased (up to 60 min), the redness of the juice increased, and then, when 60 min were exceeded, such redness decreased its intensity due to the degradation of lycopene. Other researchers have reported that tomato juice processing is one of the factors involved in lycopene (carotene responsible for the red color of tomatoes) content changes [70].

To obtain the juice, other procedures have been used, such as the pressing of the fruit. Grewal et al. employed an inverted ram hydraulic press to obtain apple juice from the entire fruit, and they obtained values of alcoholic and acetic fermentation yields of 94.64% and 83.87%, respectively, with a delightful aroma and flavor in the final vinegar [61]. When the fruit presents a softer texture, such as sour cherry, simply employing a hand press could be enough to obtain the juice, after removing the stems and core. Özen et al. did not find significant differences in the bioactive components and antioxidant activities found in vinegars that were produced from fresh sour cherry juice and concentrated sour cherry juice. However, the sour cherry vinegar produced using the concentrate juice was more prominent in terms of volatile aroma compounds, although both possibilities produced desirable aroma compounds [44]. Other authors, for the production of pomegranate vinegar, pressed the fruit with cotton filters after removing peels and mesocarps. In this case, a yield of 86.5% was obtained for alcoholic fermentation and 76.7% for acetic fermentation, and functional condiment was obtained, on the basis of its content in phenolic constituents, antioxidant activity, and attractive red color [71]. A peeling off procedure has also been employed to facilitate obtaining the juice by mechanical pressing in the production of mango vinegar, and a higher yield value of the transformation of ethanol to acetic acid was obtained (93%) [72]. Pressing has been successfully employed with other fruits such as black raspberry [73,74], cherry [75], or apricot [76], for obtaining the juice, and it is one of the most employed procedures for the treatment of the raw material in the production of fruit vinegars, due to its simplicity, because it produces high fermentation yields, preserves the bioactive composition of the fruits, and the final vinegars present positive sensory characteristics.

Finally, another possibility is the use of enzymes to obtain the juice. Usually, not all the sugars present in pineapple residues can be used in full for fermentation because pineapple peel and core contain a high amount of insoluble fiber-rich fraction, and therefore, a saccharification procedure should be employed. Roda et al. [41] employed an enzymatic digestion with different enzymes (cellulolytic, amylolytic, and invertase) after either a hydrolysis or a physical treatment of pressure (10 min, 143.27 kPa) for the production of vinegar from pineapple residues (peel, core). Cellulolytic enzymes followed by invertase achieved final reducing sugars of almost 67 and 100 g/kg of fresh weight of pineapple peel and core, respectively. In addition, the use of thermostable α-amylase during the pressure pretreatment and the subsequent hydrolysis with a mix of cellulolytic and amylolytic enzymes allowed reaching about 100 and 330 g/kg of reducing sugars in pineapple peels and core, respectively [41]. These authors later developed a vinegar obtained from the juice of saccharified pineapple waste that was clear and showed no post-filtration deposits, which was in accordance with food grade requirements. The obtained acetic fermentation yield was of 81.6%, with a value of residual ethanol concentration of 0.5% (*v*/*v*). In addition, the final product presented a high number of volatile compounds and bioactive compounds, with a low level of off-flavors [40]. Other physical pre-treatments such as microwave heating, boiling, cooking at high pressure with a pressure cooker, or with an autoclave have also been employed, among which the autoclave was the most efficient one [77]. However, when not the wastes, but the pineapple pulp is employed, just a chopping and blending procedure is enough to prepare the raw material for vinegar production. In addition, in this case, the bioactive components from the fruit are present in the pineapple vinegar, and it presents such a high antioxidant activity that it has been employed to reverse the paracetamol-induced liver damage in mice [78].

As it can be seen, there are multiple options for the juice extraction from the fruit. Those procedures in which the pulp is in close contact with the skin of the fruit such as crushing or milling would provide the juice with a higher amount of bioactive components, which are usually present in the peel. Therefore, this will help to preserve the healthy properties of the final vinegar. However, depending on the fruit employed, other solutions could be better employed, such as peeling and pressing, in order to facilitate the further process of clarification of the juice, or to avoid the contact with the peel and the extraction of substances with bitter taste, such as essential oils in citrus fruits. In that case, typical fruity aromas (mainly coming from fruit peel) could be also decreased in the final product.

## 4. Fermentation Processes

Vinegar is produced through a two-stage fermentation process, the first being the conversion of fermentable sugars into ethanol by yeasts, generally *Saccharomyces* species, and the second being the oxidation of ethanol by bacteria, generally *Acetobacter* species. Fermentation is a key process in the production of fruit vinegars, during which many volatile compounds, polyphenols, and organic acids, among others, are modified through chemical and microbial actions.

### 4.1. Alcoholic Fermentation

After the raw material preparation, the alcoholic fermentation plays a crucial role in vinegar production. Fermentation is an ancestral technique for the preservation of food and is considered a simple, natural, and valuable biotechnological process. The advantage of this technology lies with the maintenance and/or improvement of the safety, nutritional, sensory, and shelf-life properties of food products from plants [79].

°Brix grades are closely related to alcoholic fermentation, as they reflect the content of sugars, which will determine the alcoholic grade that can be obtained, and this depends on the raw material used as well as on the culture of the microorganisms used for the fermentation process. Table 1 lists the fruit used as raw material and the °Brix degrees that fruit juices are expected to have [80].

Fermentation time is also a variable parameter in this process. Different fermentation times have been described in the scientific literature for musts from different raw materials. For example, in the case of cranberry as a starting fruit, Da Silva Fonseca et al. [65] set the alcoholic fermentation time at 125 h and used the commercial strain *Saccharomyces cerevisiae bayanus*; and Yan et al. [81] used *S. cerevisiae AS2.316* yeast for 192h to ferment premier Rabbiteye cranberry juice. However, longer times have been described, such as in the case described by other authors [82] who produced wine from Brigitta blueberry using a fermentation period of 35 days at 13 °C and the strain *Saccharomyces cerevisiae*. *bayanus*. Generally, the fermentation time depends on the fruit used, its sugar level, and the physical state in which it is presented (juice, crushed, chopped, etc.). It could also be affected by the concentration of microorganisms, the sugar content, or the fermentation temperature. Similarly, the alcoholic fermentation can be carried out by spontaneous fermentation or using a starter culture, which also affects the duration of the process and the properties of the final product [30].

There are other parameters that could also affect the alcoholic fermentation, such as the fermentation temperature, the composition of the substrate, the tolerance to alcohol by the yeast used, the pH, or the sugar concentration [83]. Usually, by increasing the fermentation temperature, the fermentation rate increases. However, a much higher temperature could inhibit the growth of the microorganisms and, therefore, affect the fermentation rate. Torija et al. [84] found that the maximum speed for alcoholic fermentation was 35 °C. However, temperature tolerance is also dependent on sugar concentration. Other authors found that the fermentation of molasses at 35 °C was possible at 20% of sugar concentration but not at 22% [85]. The pH of the medium is also an important parameter that affects cell growth and fermentation efficiency. Usual values of pH are between 3.5 and 5.5, depending of the fruit employed. Moreover, not all yeasts have the same tolerance to ethanol, and this fact could also affect the alcoholic fermentation process [86]. On the other hand, increasing sugar concentration will increase the osmotic pressure and viscosity of the medium and would inhibit yeast growth and ethanol production [83]. Usual values of sugar content in the medium for a good fermentation rate should not exceed 20%. To increase the alcoholic content, avoiding the inhibition of the fermentation by substrate, a second addition of sucrose could be done after the initial level of sucrose has been consumed by microorganisms [80,87]. By this procedure, a higher content of acetic acid could be achieved in the final vinegar.

It should also be noted that the alcoholic fermentation of the sugars generates a number of by-products including glycerol, which after ethanol is the alcohol that is most widely used by acetic acid bacteria. However, the excess of glycerol may reduce the ethanol yield during wine production. Factors such as temperature, aeration, sugar concentration, and osmotic stress could influence the production of glycerol during alcoholic fermentation [88]. Some authors have found differences in the ethanol/glycerol ratio produced under static and dynamic (agitation) alcoholic fermentation conditions, with a maximum ratio of 29 after 27 h in the agitated fermentation toward 47 after 45 h in the static one. Glycerol is a non-aromatic compound. Nevertheless, it can significantly contribute to wine’s quality, providing sweetness and fullness [89]. Glycerol acts as a carbon source for *Acetobacter* species and protects them from hard conditions such as high pH situations. In this way, acetic acid bacteria can survive and energetically grow for a long time in a glycerol-containing medium. Acetic acid bacteria can employ glycerol as a carbon source and transform it into dihydroxyacetone (DHA) [90]. Therefore, the ethanol/glycerol ratio is a key parameter for the vinegar quality. In particular, a high ratio should be adequate for an optimal acetification step. Some of the scientific research studies that include the ethanol/glycerol ratio were performed by Lea [91] where 0.23–0.56% glycerol content is present in apple vinegar, while in other study [75] on cherry vinegars, glycerol content levels were lower.

In relation to the differences found for glycerol/ethanol ratio according to an agitated or static alcoholic fermentation, wines from agitated/static process presented 5.73 and 6.81% (*v*/*v*) of alcohol content, and the values of total acidity were 3.9 and 4.4 g/L, respectively, with volatile acidity of 1.1 and 1.2 g of acetic acid/L, for the agitation and static processes. A pH value of 4.0 for both fermentation processes was found. The agitated fermentation showed a higher ash content and total dry extract, 11.25 g/L toward 8.63 g/L in the static process. Regarding volatile compounds, in general, the agitated process produced a wine with a lower volatile content: ethyl acetate, 18.1 mg/L for the agitation and 23.2 mg/L for the static process; acetaldehyde, with values of 22.1 and 89.9 mg/L for agitation and static fermentation, respectively; furfural; some alcohols such as methanol and isoamyl alcohol; etc. In summary, the productivity in the agitation process was higher than in the static, and shorter times were required [92].

In relation to the influence of a possible agitation during alcoholic fermentation, scarce literature can be found in which both methodologies, static and dynamic conditions, were compared for fruit wines. Coelho et al. carried out a study in which the production of four fruit wines, orange, mango, cherry, and banana, were optimized. All the fermentation studies were carried out under agitation conditions. The alcoholic fermentation did not affect the fruits’ antioxidant activity, and the orange and cherry vinegars showed the highest antioxidant activities in concordance with the values found for this parameter in the raw materials. In this case, cherry vinegars were the most acceptable ones from a sensory point of view [93].

#### Microbiological Aspects of Alcoholic Fermentation on Fruit Vinegars

- Spontaneous Alcoholic Fermentation

Numerous studies have been carried out on natural or spontaneous alcoholic fermentation processes [29,31,52,53,74,94]. During natural fermentation, the changing environmental conditions favor the proliferation of the most suitable native microbiota for the processing of the raw material. The stricter the growth conditions, the greater the selective pressure exerted on the native microorganisms. Song et al. [74] produced black raspberry vinegar using strains of native yeasts for the alcoholic fermentation. These native yeast strains showed improved growth and an increased ethanol production rate in comparison with other commercial yeasts. In addition, some differences in terms of physical–chemical properties of the final vinegars produced could be observed depending on the type of yeast used for the alcoholic fermentation, as well as an increased antioxidant capacity when using native yeasts.

The use of native yeasts and spontaneous alcoholic fermentation also have some drawbacks, such as the higher risk of contamination with other undesirable microorganisms, the uncertainty about the properties of the obtained product, the usual longer periods employed for the beginning of the fermentation, or the possibility of having a lower population of yeasts, which could interfere with the fermentation process. However, Hidalgo et al. [53] obtained the alcoholic substrate for the elaboration of persimmon vinegar both by natural fermentation and through inoculation of *S. cerevisiae*, and in both fermentations, the same yeast population was reached: 10^8^ cells/mL. Similar values of yeast population were found in the natural fermentation of other fruits such as gabiroba [95], apple [96], strawberry [97], or pineapple [94] when used for the production of alcoholic beverages. 

In most spontaneous fermentations, a microbial succession takes place, and quite often, lactic acid bacteria and yeasts dominate at the beginning of the process. Generally, it is yeasts other than *Saccharomyces* that start the spontaneous alcoholic fermentation, until finally, *S. cerevisiae* is the one to dominate the process [53]. These consume sugars and produce ethanol, which inhibits the growth of many bacteria species, which results in a longer shelf life of the products. This phenomenon has been described for gabiroba wine [95], although in fermentations that yield a low final alcohol content, *Saccharomyces* may not always appear [94]. In the spontaneous fermentation of persimmon, *P. guilliermondii*, *H. uvarum*, *Z. florentinus* and *Cryptococcus* sp. were isolated during the entire fermentation process [53]. Non-*Saccharomyces* yeasts usually present a higher diversity when spontaneous fermentations are carried out, since inoculation with selected yeasts usually reduces the growth of native yeasts [98]. The presence of non-*Saccharomyces* yeasts is related to the sugar concentration or the presence of organic acids in raw material, and these yeasts may alter the volatile profile of the wine produced, compared to that produced mainly with *Saccharomyces* strains [53]. Although non-*Saccharomyces* yeasts are usually employed in alcoholic fermentation to reduce alcohol content of wine [99], and they can affect the quality parameters during wine fermentation [100], scarce literature exists regarding the effect of these species on the quality of fruit vinegars. These kinds of yeasts are usually common when subtracts with high sugar concentration (30–50%) and low values of pH (<3) are employed, such as in the case of traditional balsamic vinegar [101]. Some authors found that some non-*Saccharomyces* yeast strains such as *Candida ethanolica*, *Pichia membranifaciens*, and *Saccharomycodes ludwigii* were present in conventional and organic apple cider vinegars, presenting a high acetic acid resistant, and the differences in the composition of microbiota could influence the chemical composition and sensorial quality of vinegars [102]. Other studies have shown that non-*Saccharomyces* yeast species such as *Candida* and *Saccharomycodes* appeared during the initial and middle stages of acetification for wine vinegar or kombucha vinegar, often showing more beneficial effects with positive metabolic activities [103]. In addition, Kawa-Rygielska et al. demonstrated that the use of *Saccharomyces* non-*cerevisiae* strains such as *Saccharomyces bayanus* to carry out alcoholic fermentation significantly increased the content of biologically active compounds and antioxidant activity in cornelian cherry vinegars [75].

- Alcoholic Fermentation using a Starter Culture

A starter culture is defined as a preparation containing a large number of cells of a particular microorganism that is added to the raw material to trigger and lead the fermentation process of a food product. This is a frequent practice to obtain the alcoholic medium for vinegar production, since it ensures the quality and reproducibility of the final product [104], and it also shortens the fermentation time and increases the safety of the product [29]. As an example, in a study on strawberry and persimmon vinegar production, the alcoholic fermentation took place more rapidly when yeast inoculation was used, since the lag phase was shorter [52,53]. Numerous studies are available in the literature in which the most commonly used yeast for alcoholic fermentation is *Saccharomyces cerevisiae* [28,37,45,47,48,49,56,63,65,105,106,107,108], but others can also be found where mixed cultures are used, such as the mixture of *Saccharomyces cerevisiae* with *Lactobacillus plantarum* for the production of citrus vinegar [38]. In this study, the contents of sweet and umami free amino acids were higher when the mixture was employed, and flavor groups such as esters, alcohols, and aldehydes also significantly improved. From an organoleptic point of view, citrus vinegar produced with the mixture showed higher intensity for sweet and umami attributes, as well as flowery and fruity ones. Moreover, the utilization of mixed culture in alcoholic fermentation significantly improved the antioxidant activity in citrus vinegar [38].

During alcoholic fermentation, besides the production of ethanol, a large number of chemical compounds are normally modified. For example, Su and Silva [67] made rabbiteye cranberry vinegar using *S. cerevisiae* for the alcoholic fermentation, and this fermentation reduced the total anthocyanin and polyphenol content of the by-products, but it did not affect the antioxidative activity. In contrast, Kong et al. [64] found no significant differences in polyphenolic content and antioxidative activity when alcoholic fermentation was carried out with added to dry yeast for the production of papaya vinegars. This modification of substances might be influenced by the type of fermentation used (spontaneous or with starter culture). Úbeda et al. [30] when producing strawberry vinegars showed that the wines produced using starter culture presented half the anthocyanin content, in comparison to those obtained by spontaneous fermentation. Regarding the modification of the volatile composition during alcoholic fermentation, Úbeda, Callejón, et al. [56] found that the yeast strain used influenced the production of acetaldehyde and higher alcohols during the alcoholic fermentation of strawberry or persimmon for the production of vinegars. In another study on the production of gabiroba wine [95], the inoculated yeasts produced larger amounts of ethanol and higher alcohols compared to those obtained using native yeasts. In relation to organic acids, several authors have found important variations of these compounds during the alcoholic fermentation when a starter culture for the production of fruit vinegars was used [64,109]. Concretely, the concentration of lactic acid increased and was accumulated during alcoholic fermentation, whereas other acids, such as malic or citric acid decreased significantly in the production of wine from peach [109]. Ascorbic acid content can also increase during alcoholic fermentation because yeast could produce precursor antioxidant molecules such as D-erythroascorbic acid [110]. Lorenzini et al. [111], in the fermentation of apple juices with *Saccharomyces* and non-*Saccharomyces* strains, observed that the malic acid content was similar in all ciders. The content in acetic acid was low in cider produced by the two *Saccharomyces* strains, *T. debrueckii TD291* and *Z. bailii ZB3*, while *S. bacillaris YR21* was the largest producer for this organic acid. Succinic acid is the main acid produced by yeasts during alcoholic fermentation. A high amount of this acid could influence negatively on the final quality of fruit wines. Duarte et al. [112] found similar concentrations of ethanol, glycerol, and malic acid for three raspberry wines obtained with three yeast strains (*CAT-1*, *UFLA FW 15*, and *S. bayanus CBS 1505*), whereas the wine fermented by *UFLA FW 15* showed the highest amount of succinic acid (7.9 g/L).

Other authors [64] for the elaboration of papaya vinegar carried out a pasteurization process before and after the alcoholic fermentation in order to eliminate any possible microorganisms and prevent any undesired modifications of the sample’s content. In this case, they used active dry *S. cerevisiae* (ADS) yeast in powder form, which under anaerobic conditions and at an incubation temperature of 30 °C for 7 days allowed the production of papaya wine. 

Alternatively, the starter culture can be achieved by cell immobilization. Encapsulation is the most often used immobilization method. This method consists of confining the intact active cells within a specific region. Some of its advantages are the following: stimulation of the production, prolongation and excretion of secondary metabolites (e.g., aromatic compounds), continuous cell recovery and reuse, and protection against unfavorable environments, among others [113,114].

Another important advantage that this technology provides consists of the reduction of processing costs and the possibility of customizing the properties of the product of interest, such as improving its organoleptic characteristics and safety, or generating specific functional properties such as the increase of antioxidant capacity derived from the polyphenolic content, melatonin production released by yeasts, or probiotic and immunomodulatory properties. Immobilization mimics the cellular aggregation phenomenon that normally occurs when microorganisms grow in natural environments. Several substances have been investigated to be used as an aid for immobilization. Leonés et al. [80] used two types of commercial yeasts: *Saccharomyces cerevisiae* AWRI 796 and *Saccharomyces cerevisiae var. bayanus* for the alcoholic fermentation that was required for the production of lemon vinegar. For each strain, they carried out both submerged and immobilized culture in alginate spheres. The best conditions for the alcoholic fermentation were obtained when *Saccharomyces cerevisiae var. bayanus* was used in a submerged culture, since a higher alcoholic degree was reached. This could be probably explained by the fact that when yeasts move freely in the medium, a larger amount of nutrients is at their disposal than when they are immobilized and, therefore, this improves the performance of the process.

### 4.2. Acetic Fermentation

Once the sugar has been transformed into ethanol, the next fermentation that takes place in the process to elaborate fruit vinegars is the acetic fermentation, which consists of the oxidation of the alcohol into acetic acid. This is an oxygen-dependent reaction, and, therefore, as the amount of oxygen decreases with the alcoholic fermentation, once the sugar is depleted, the oxygen concentration must be increased again for the acetic fermentation to take place.

The dynamic changes in the microbial community during acetic fermentation are different from those taking place during other stages of fermentation [115]. The high concentration of ethanol at the initial stages and the high acidic conditions of the middle and final stages suggest that most of the bacteria present are acetic acid bacteria. Therefore, the biotransformation of ethanol into acetic acid is usually performed by that type of bacteria. When bacteria use acetic acid as a carbon source, a peroxidation of the acetate can occur, which in turn leads to over-oxidation and to the formation of carbon dioxide and water [60]. If there are no losses due to evaporation or over-oxidation, the total concentration—the sum of the ethanol concentration (% *v*/*v*) plus the total acidity (% *w*/*v*)—should remain constant over the acetification process.

As already mentioned, the metabolism of acetic acid bacteria is aerobic; however, they can survive under anaerobic conditions or with very low oxygen concentrations since they have the possibility to use quinones instead of oxygen as the final electron acceptor [116,117].

On the other hand, it is also known that the concentration of ethanol could exert an inhibitory effect on acetic acid bacteria when it is above 50 g/L (approximately 6% *v*/*v*), this being more pronounced in discontinuous processing [118]. For this reason, there are studies, such as the one by Davies et al. [119] on the production of orange vinegar, where orange wine, which had an alcoholic content of 13–14%, was diluted in order to facilitate the action by acetic acid bacteria. The dilution caused a variation in the concentration of nutrients, and a solution with minerals and a source of nitrogen had to be added.

The optimal growth temperature for these bacteria is between 25 and 30 °C, while the maximum temperature that they can tolerate may reach 40 °C [120,121]. Since the oxidation of ethanol into acetic acid is an exothermic reaction, excessive temperatures could destroy acetic bacteria and increase the evaporation of volatile compounds, such as ethanol or acetic acid. If this is the case, the resulting vinegar quality and yield would be affected. In order to prevent these negative effects, the fermenter should be equipped with heat dissipation systems, such as cooling coils [122]. It has been proven that by slightly increasing the fermentation temperature, the productivity of the process can be enhanced, even though it could favor the oxidation processes and the loss of aromatic components. However, the use of temperature gradients during the acetification process is proposed as a suitable solution, which would slow down the process and at the same time would prevent these previously mentioned inconveniences. Fregapane et al. [123] observed that a variation of only two centigrade degrees at the beginning of the acetic fermentation (32 °C) and subsequently decreasing the temperature to 30 °C produced a 15% increment in acetic acid production and a shortening of the processing time from 29 hours to 24.5 hours in comparison with an isothermal fermentation at 30 °C.

Acetic acid bacteria are a wide and well-distributed group that can be found in fruits, flowers, honey, soil, juices, and fermented beverages, among others [124]. In terms of taxonomy, there are currently 19 genera classified under acetic acid bacteria [125]. The exploitation of those bacteria has a long history in fermentation processes, and nowadays, they represent an emerging field in biotechnological applications such as the biosynthesis of chemical products or food science. Their most recognized application at present is the production of vinegar, and the species of the genera *Acetobacter*, *Gluconobacter*, and *Gluconacetobacter* are the most commonly used for this purpose. Generally, *Acetobacter aceti* is the most widely used bacterium in the vinegar industry, since it is the one that usually starts the acetic fermentation [43], while *Gluconobacter* can provide a slightly different taste to vinegar due to the production of gluconate [126]. However, the production of D-gluconic acid has also been detected in acetic acid bacteria such as *Acetobacter syzygii* [127]. Hidalgo et al. [53], during the production of persimmon vinegar, identified bacteria such as *Acetobacter malorum*, *Gluconacetobacter saccharivorans*, *Acetobacter pasteurianus*, *Acetobacter syzygii*, *Gluconacetobacter intermedius*, or *Gluconacetobacter europaeus*, among others. In another study carried out using the acetic acid bacteria isolated from blueberries, several different genera of these were identified through biochemical tests (*Acetobacter*, *Gluconobacter*, *Asaia*, *Gluconacetobacter* and *Swaminathania*) dependent on the different varieties of blueberries used for the experiments [31].

However, the use of starter cultures is far from being applied at a large scale, mainly for two reasons: firstly, because it would not be economically profitable, since vinegar is a generally inexpensive product and elaborating it with a starter culture would increase costs; and secondly, because the nutritional requirements by acetic bacteria present considerable difficulties for their cultivation and conservation in laboratories [1]. Nevertheless, there have been studies that have used bacterial inocula such as *Acetobacter malorum* for the production of strawberry vinegar [52]. However, these authors observed that this strain of bacteria was displaced by *Gluconacetobacteria* when the acetic fermentation took place in wooden barrels. Another example would be the study carried out by Boonsupa [58], who spent 15 days experimenting with the acetic fermentation of blackberry, blueberry, and raspberry wines inoculated with *Acetobacter pasteurianus*. The same authors carried out the fermentation of banana vinegar using the same bacterial strain [60].

Although it is still a field in which scarce scientific literature is found, the use of acetic acid bacteria as starter of the fermentation would present several advantages, compared to spontaneous acetic fermentation. According to Hidalgo et al. [52], the use of starter cultures induced a fast beginning of the acetification and provided the appropriate conditions for the correct development of the process, avoiding stuck acetification. Moreover, in the inoculated processes, the final acidity of vinegars seems to be higher. Concretely, in this study, the samples inoculated with *Acetobacter cerevisiae* reached higher acidity values (from 6.6% to 6.9% (*w*/*v*)) in shorter times than those with spontaneous fermentation (5.5% *w*/*v* after 28 days).

Úbeda et al. [56] reported differences of 2 acetic degrees between spontaneous and inoculated fermentation of strawberry vinegars. In addition, the study of the aromatic fraction demonstrated that inoculated acetification carried out in wood barrels yielded vinegars with a better aroma profile. The same authors described significant differences in ethyl acetate content, which increased from wine to vinegar when starter cultures were employed, with values from 83 to 682 mg/L, whereas for spontaneous processes, it diminished, due to hydrolysis phenomena, showing values from 45 to 483 mg/L [56]. This could affect the final organoleptic properties of the vinegars, because ethyl acetate presents a strong “glue” odor, so this character would be more intense when inoculated fermentations are carried out.

Higher alcohols and methanol also showed differences between spontaneous fermentations and fermentations performed with selected acetic acid bacteria. Regarding the former ones, their consumption was higher in those vinegars fermented with starters, whereas methanol showed a larger decrease in the spontaneous fermentation. Finally, it is worth mentioning that there are some studies in which high contents of polyphenols and antioxidant activities have been reported in fruit vinegars produced with *A. pasteurianus* [58,60]. Therefore, it seems that by the careful selection of the bacteria strain employed in the acetic fermentation, some bioactive components could also be promoted to the final product.

As can be seen, the composition of fruit vinegars depends on the acetic acid bacteria strain that carries out the fermentation. In addition, faster fermentations are usually obtained when starters are employed. Therefore, a comprehensive control can be obtained if starters are employed in the production of fruit vinegars. In this way, products with the expected composition and organoleptic properties by producers and consumers can be obtained. The type of microorganism used both, in alcoholic and acetic fermentation, affects the final characteristics of the vinegar produced. However, scarce information about different acetic acid strains and the consequences of their use in the production of fruit vinegars is available. That could be a future research subject in order to obtain fruit vinegars even from the same raw material but with different sensory properties, increasing, in this way, the variety of acetic products that can be commercialized.

#### Acetification Systems

There are basically two acetification methods: surface and submerged cultivation systems.

In the surface culture method, acetic acid bacteria grow abundantly on the surface of the medium, at the liquid–gas interface, where the highest concentration of oxygen is present. This is considered a static method, where the presence of bacteria at the interface is limited for physical reasons. There are numerous investigations where this method of acetification has been applied to the elaboration of fruit vinegars [28,32,37,44,46,49,58,66,67,73,106,128,129]. The acetic acid values obtained using this method of fermentation on surface cultures are usually not too high. For instance, Özen et al. [44] used this surface cultivation method for the elaboration of cherry vinegars, both from fresh juice and from concentrate, achieving acidity values above 4.6%. Cejudo-Bastante et al. [37], during the elaboration of orange vinegars by means of surface cultivation, obtained similar values of acidity (around 4%), and the final vinegars presented good organoleptic characteristics. On the other hand, Fatima and Mishra [32] obtained acidity values between 5% and 6% in coconut water vinegars and slightly less in banana skin vinegars. In addition, the times employed for the fermentation by means of surface culture are usually relatively long. Fermentation times as long as 144 days have been reported for the acetic fermentation of black raspberry vinegars [73], although shorter periods have also been reported, such as 45 days for plum vinegars [46], 30 days for cherry vinegars [44], or 15 days for berry vinegars [58]. A reference has been found in which the Schützenbach method was used for the elaboration of blueberry vinegars [31]. This method, which allows an acceleration of the process, uses wood shavings to support the bacteria, and the liquid is pumped over the shavings to increase the oxygen supply. Using this system, the process of acetification was accelerated to 9–24 days compared to the traditional method of surface cultivation that required more than 30 days.

**Figure 4:**
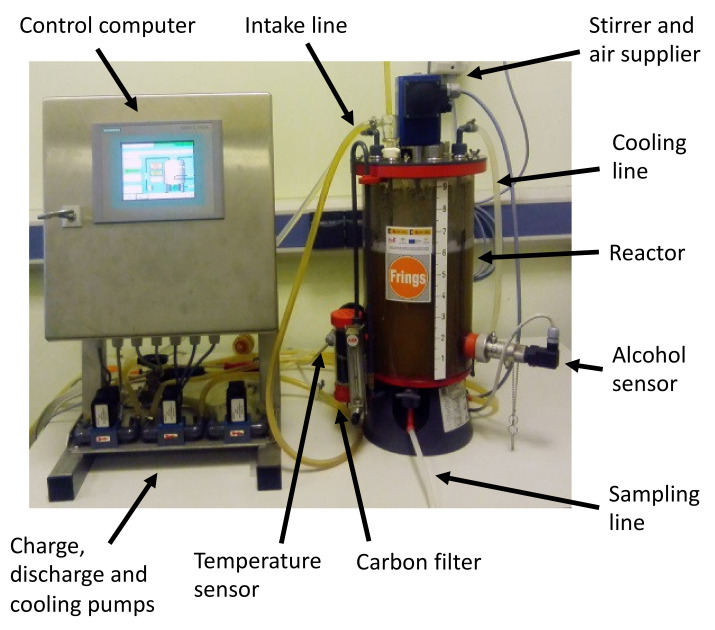
Typical system for submerged culture fermentation.

On the other hand, the methods of fermentation using submerged culture are based on the presence of a culture of bacteria freely submerged within the liquid to be fermented. Air is constantly supplied (either on its own or enriched with oxygen), and no additional support is provided to the bacteria [130]. For these methods, acetifiers are used, which are usually automated and provide a high flow of oxygen (Figure 4). Therefore, these methods usually present higher yields than those obtained through surface culture fermentation [131].

However, surface cultivation methods have traditionally been considered as being suitable for the production of quality vinegars. Molelekoa et al. [129] used surface and submerged culture for the production of marula vinegar (a fruit from South Africa). When surface culture was used for the fermentation process, the final product had a higher antioxidant and anti-radicals power.

The investigations that have used the method of elaboration of fruit vinegars by means of submerged culture are also very numerous, of which orange has been the most commonly used fruit [28,42,119], followed by pomegranate [45,71]. Nevertheless, other research studies have been found on strawberries [62], persimmon [48], peach [109], tomato [132], lemon [80], apricot [76], and marula [129].

Fermentation times when using submerged culture are usually much shorter than those used in surface culture. Cejudo-Bastante et al. [28] compared the two systems of acetification for the elaboration of orange vinegar, and 6 weeks was used for the fermentation in surface culture, as opposed to 22 hours used for the fermentation in submerged culture. The constant supply of oxygen during the whole process is vital, since these species are strictly aerobic, and an interruption of the air supply may result in the death of the culture [133]. At the beginning of the process, the level of the air flow must be maintained at low levels, around 1 L of O_2_/h L of substrate, to favor the reproduction of the bacteria. This should be increased to values around 7.5 L of O_2_/h L of substrate after the acetic fermentation has begun [28,80,132].

Many compounds are normally degraded during the acetic fermentation process in vinegar production, and this fact is more pronounced when the submerged culture method is used. This is explained by the increased yield of the process. In an experiment in which strawberry vinegars were developed, 91% of the anthocyanins were lost during the acetic fermentation, compared to just 19% losses during the alcoholic fermentation [62]. In another investigation with pomegranate vinegars, it was observed that volatile and polyphenolic compounds increased during the alcoholic fermentation but decreased during the acetic one [45]. Another study reported reductions around 60% of the polyphenolic content in pomegranate vinegars compared to the starting juice [71]. Although it is difficult to avoid these losses of bioactive compounds during the acetification process due to the biochemical nature of the process, it could be attenuated when surface culture is employed. When operating with submerged culture, a high amount of oxygen is supplied in order to accelerate the oxidation reaction of alcohol into acetic acid. For an industrial tank holding 25,000 L and operating at an acetification rate of 0.2% acetic acid · h^−1^, it will require about 20,000 L of oxygen (at 20 °C, 1 atm) per hour (i.e., 26.7 kg O_2_·h^−1^) [131]. Taking into account that air is used to supply the oxygen and that only 60–90% of the oxygen supplied is employed to oxidize ethanol, the amount of air required at 20 °C and 1 atm would be 100,000–150,000 L·h^–1^. This high amount of air would also provoke the acceleration of other oxidative reactions of bioactive compounds and therefore would favor some degradation processes when submerged culture is employed. As it has been commented previously, the selection of specific strains of acetic acid bacteria that favored the production of bioactive components could help diminish these losses. For example, the production of D-gluconic acid, which has been demonstrated to be an interesting compound with healthy properties [134], could be favored by the specific strains of acetic acid bacteria employed, such as *Gluconobacter japonicus*, or *Gluconobacter oxydans* [135]. Usually, *Gluconobacter* strains are generally more ketogenic than *Acetobacter* strains. Thus, *Gluconobacter* strains oxidize a broader range of substrates compared to *Acetobacter*, such as alcohols, sugars, sugar acids, or sugar alcohols, and therefore, the corresponding oxidation products are accumulated in the medium [136]. For the preservation of the volatile fraction, De Ory et al. [137] proposed an acetic acid fermentation reactor equipped with a closed gas recycling system that prevents any loss of volatile compounds due to evaporation. With this system, the evaporative losses were reduced to 0% during the acetic acid fermentation process.

Three modes of operating the fermenter are available when working in submerged culture: discontinuous, semi-continuous, or continuous [131]. Virtually all the research related to the development of fruit vinegars uses the semi-continuous mode, in which the whole fermenter is not discharged when the process of acetification has been completed. Instead, only part of it is discharged, while another part is used as the starter for the next fermentation cycle, which speeds up the process [138]. The discharge volume may vary, but it is usually between one-half and two-thirds of the fermenting volume. For example, Hornedo-Ortega et al. [62] developed strawberry vinegar by operating in a semi-continuous mode and discharged about 73% of the fermenting volume. On the other hand, Cejudo-Bastante et al. [132] used this method for the production of tomato vinegars, performing a 66% discharge, the same value of Leonés et al. [80] for the production of lemon vinegars.

Regarding the effect of acetification system on sensory properties of fruit vinegars other than grapes, only one reference has been found in the literature, in which both systems were employed for the production of orange vinegar and sensory evaluation was carried out [28]. The submerged culture produced more pungent vinegars, with higher scores of the descriptors “fruity”, “floral”, and “glue”, and with better values of “general impression”, compared to vinegars obtained by means of surface culture. In another work concerning Turkish grape vinegar, the authors obtained higher values of acidity and contents of volatile compounds with the surface culture method [139]. However, regarding sensory characteristics, these authors reported significant differences only for ethyl acetate odor and aromatic intensity (higher for surface culture); the rest of descriptors were significantly similar for both acetification systems.

## 5. Conclusions

The production of fruit vinegars is one of the most commonly used options as an alternative for the exploitation of existing fruit surpluses, thus reducing the economic and environmental impact generated by the fruit industry. As it has been proven, raw material and its treatment are key factors for the elaboration of quality products, since the final chemical properties of vinegar depend on them. The type of microorganism used, both in alcoholic and acetic fermentation, also affects the final characteristics of the vinegar produced, while the acetification system used (surface or submerged) is another very important factor with regard to the physicochemical properties of the fruit vinegars that can be obtained.

Even though there are numerous scientific articles related to the elaboration of this type of vinegar, more experiences are still needed to determine the ideal conditions for the elaboration of fruit vinegars according to optimized biotechnological processes that result in a higher profitability of the product. Particularly, since commercial initiators to start the acetic fermentation are not used, this may lead to problems in the production of the vinegars and result in economic losses. If only such starters were commercialized at competitive prices, this would help to speed up the start of acetic fermentation, to prevent pollution and to shorten processing times. Another promising line of research is related to the use of thermotolerant bacteria that would allow acetic fermentation to be carried out at higher temperatures. In this way, cooling costs could be saved and vinegars could be produced in shorter times. However, the possible loss of aromatic substances is still an important drawback that should be taken into account when carrying out such studies.

Another future research line could be the use of innovative technologies such as high hydrostatic pressure, ultrasound, microwaves, pulsed electric fields, and so on, and their effects in the production of fruit vinegars with high standards of quality. Some of them have been tested for the production of vinegars from grapes, and the results have been promising.

## Figures and Tables

**Table 1:** Minimum °Brix expected from the different fruit juices.

Common Name	Botanic Name	°Brix
Apple	*Malus domestica*	11.2
Apricot	*Prunus armeniaca*	11.2
Banana	*Musa x paradisiaca*	21.0
Blackcurrant	*Ribes nigrum*	11.0
Grape	*Vitis vinifera*	15.9
Grapefruit	*Citrus x paradisi*	10.0
Guava	*Psidium guajava*	8.5
Lemon	*Citrus limon*	8.0
Mango	*Mangifera indica*	13.5
Orange	*Citrus sinensis*	11.2
Passion Fruit	*Passiflora edulis*	12.0
Peach	*Prunus persica*	10.0
Pear	*Pyrus communis*	11.9
Pineapple	*Ananas comosus*	12.8
Raspberry	*Rubus idaeus*	7.0
Cherry	*Prunus cerasus*	13.5
Strawberry	*Fragaria x ananassa*	7.0
Tomato	*Lycopersicon esculentum*	5.0
Tangerine	*Citrus reticulata*	11.2
